# Outcomes in patients not conveyed by emergency medical services (EMS): a one-year prospective study

**DOI:** 10.1186/s13049-022-01023-3

**Published:** 2022-06-13

**Authors:** Erik Höglund, Agneta Schröder, Magnus Andersson-Hagiwara, Margareta Möller, Emma Ohlsson-Nevo

**Affiliations:** 1 Faculty of Medicine and Health, University Health Care Research Centre, Örebro University, Box 1613, 701 16 Örebro, Sweden; 2 Department of Health Sciences in Gjøvik, Faculty of Medicine and Health Sciences, NTNU-Norwegian University of Science and Technology, Gjøvik, Norway; 3 Faculty of Caring Science, Centre for Prehospital Research, Work Life and Social Welfare, University of Borås, Borås, Sweden; 4 Department of Surgery, Faculty of Medicine and Health, Örebro University, Örebro, Sweden

**Keywords:** Ambulance, Emergency medical services, Non-conveyance, Non-transport, Outcome measures, Triage, Quality

## Abstract

**Background:**

The decision to not convey patients has become common in emergency medical services worldwide. A substantial proportion (12–51%) of the patients seen by emergency medical services are not conveyed by those services. The practice of non-conveyance is a result of the increasing and changing demands on the acute care system. Research focusing on the outcomes of the decision by emergency medical services to not convey patients is needed.

**Aim:**

The aim was to describe outcomes (emergency department visits, admission to in-hospital intensive care units and mortality, all within seven days) and their association with the variables (sex, age, day of week, time of day, emergency signs and symptoms codes, triage level colour, and destination) for non-conveyed patients.

**Methods:**

This was a prospective analytical study with consecutive inclusion of all patients not conveyed by emergency medical services. Patients were included between February 2016 and January 2017. The study was conducted in Region Örebro county, Sweden. The region consists of both rural and urban areas and has a population of approximately 295,000. The region had three ambulance departments that received approximately 30,000 assignments per year.

**Results:**

The result showed that no patient received intensive care, and 18 (0.7%) patients died within seven days after the non-conveyance decision. Older age was associated with a higher risk of hospitalisation and death within seven days after a non-conveyance decision.

**Conclusions:**

Based on the results of this one-year follow-up study, few patients compared to previous studies were admitted to the hospital, received intensive care or died within seven days. This study contributes insights that can be used to improve non-conveyance guidelines and minimise the risk of patient harm.

**Supplementary information:**

The online version contains supplementary material available at 10.1186/s13049-022-01023-3.

## Background

Both nationally and internationally, a considerable and increasing number of patients are being non-conveyed (12–51%) [1–5]. Non-conveyance has become a way for emergency medical services (EMS) to address the increasing and changing demand placed on overloaded acute-care systems [6, 7]. Even if non-conveyance has become an everyday practice for EMS worldwide, the decision to non-convey a patient is described as complex and involve a significant amount of responsibility [6, 8–10]. Patient-safety issues have also been raised [11, 12]. Previous research shows contradictory results regarding the EMS capability to safely non-convey patients [4, 11–13]. Despite these patient safety concerns, validated guidelines to inform the decision regarding non-conveyance in the prehospital context are still lacking [14, 15]. To describe patient safety aspects regarding EMS-initiated non-conveyance, outcome measure rates, such as subsequent emergency department visits, admission to in-hospital care units, and mortality, are described [13, 15].

Outcome measure rates for these non-conveyance decisions, based on existing guidelines, are heterogeneous and vary between contexts [16–18]. Subsequent emergency department visit, in-hospital admission, and mortality rates have been shown to vary from 4.6 to 25.8%, 3.3–12.1%, and 0.2–6.1%, respectively, demonstrating this difference according to context and reported follow-up period [13, 15]. Several factors have been described to increase the likelihood of non-conveyance, such as younger age, female gender, time of day and alcohol use [19]. Even though younger patients are more likely to not be non-conveyed, older patients are more often admitted to in-hospital care units following a non-conveyance decision [11, 19].

Since it is still unclear whether ambulance services can safely non-convey patients, guidelines for the general non-conveyance population need to be validated. For future development and validation of these guidelines, more research is needed that describes non-conveyance and what factors are associated with adverse outcomes.

## Aim

The aim was to describe outcomes (emergency department visits, admission to in-hospital intensive care units and mortality, all within seven days) and their association with the variables (sex, age, day of week, time of day, emergency signs and symptoms codes, triage level colour, and destination) for non-conveyed patients.

## Methods

This study is part of a larger project called Non-conveyance - Go to Other Level of Care (No-Go), which has the overarching purpose of describing, analysing, and developing safe non-conveyance guidelines and decision-making support systems [3, 9].

### Study design

This was a prospective analytical study that consecutively included patients who were not conveyed by the EMS. The current study follows the Strengthening the Reporting of Observational Studies in Epidemiology (STROBE) guidelines [20].

### Study setting

The study was conducted in Region Örebro county, Sweden. The region had a population of approximately 295,000. The regional EMS consisted of three ambulance departments that received approximately 30,000 assignments per year. Twelve ambulances operated 24 h per day seven days a week, and an additional four ambulances operated during the daytime and evening hours. In the studied region, there was one level-one trauma centre and two smaller hospitals. The region consisted of both rural areas and small- to mid-sized cities. Registered nurses, with three years of university education, and specialist ambulance nurses, with an additional year of university education and a master’s degree in emergency care, staffed the ambulances. The ambulances were staffed by two people. An emergency medical technician, with the equivalent of one and one-half to two years of basic education and an additional practical ambulance education, sometimes replaced one nurse or specialist ambulance nurse in the team. Only specialist ambulance nurses are employed full time on a permanent basis, making it uncommon for ambulances to be staffed with registered nurses.

### Regional non-conveyance guidelines

The regional guidelines for non-conveyance, which were implemented in 2015, allow registered and specialist nurses to independently make the decision to not convey a patient. The guideline checklist is provided as Additional file 1 and additional exclusion criterias as Additional file 2. The guidelines were designed to be restrictive and to identify as many patients as possible who might risk deterioration. The guidelines state that patients cannot require the administration of drugs, supervision, or monitoring during transport to a health care facility. Patients (or their legal guardians) also must be able to communicate and understand the decision and information provided. The nurses have the option to contact a physician at the receiving hospital who can make a decision that contradicts the recommendation based on the guidelines. According to the guidelines, recommendations can be made for patients to seek different levels of care. The lowest level of care is self-care, followed by primary health care and care in the ED reached via personal or public transport.

When the decision is made to not convey a patient, a document containing information about the assessment, recommendations regarding further health care contact, and where the patient can seek assistance if the condition worsens is created and given to the patient. The nurse can non-convey a patient even if the patient prefers to go to the hospital by ambulance. Most often, the patient is involved in the decision.

Patients can be conveyed even if, according to regional non-conveyance guidelines, they are eligible for non-conveyance. Patients can, for instance, be conveyed due to humanitarian reasons or a lack of alternate options, or if the nurse, regardless of the triage outcome, believes that the patient needs additional assessment. During the study period, region-specific guidelines were used together with the Rapid Emergency Triage and Treatment System (RETTS) [21, 22]. For non-conveyance to be considered, all vital parameters must be within the normal ranges. Children must receive the lowest triage level colour (green), and adults (≥ 18 years) must receive the lowest (green) or next lowest (yellow) triage level in the four-colour system, in which patients can be assigned green, yellow, orange, or red triage colours.

The triage system combines the patient’s signs and symptoms and the main complaint, coded with an emergency signs and symptoms (ESS) code, with the patient’s vital signs to determine the urgency level. These ESS codes and vital sign cut-offs are different for children and adults. The different urgency levels indicate the time within which the patient needs to be seen by a physician. Green and yellow triage level colours indicate that the patient does not need immediate emergency care and could wait for longer than three hours to receive an additional assessment. The orange triage level colour means that the patient has urgent medical needs but could wait for up to 20 min before being assessed by a physician. The red triage level colour means that the patient needs immediate assessment and treatment. The RETTS was initially designed for use in the ED. Neither the regional non-conveyance guidelines nor the RETTS were validated for use when making prehospital non-conveyance decisions at the beginning of this study.

### Study population

All paediatric and adult patients who were not conveyed by the EMS in the studied region between February 2016 and January 2017 were included. Patients were excluded if they refused care, examination or conveyance. Patients who were found dead at arrival were also excluded.

### Data collection

Data were obtained from handwritten medical records. All records produced during the study period and submitted according to the regional guidelines for non-conveyance documentation were included in the study. The regional non-conveyance guideline was implemented approximately one year before the start of data inclusion. Patients could be non-conveyed more than one time. A total of 163 patients were non-conveyed more than once, corresponding to 431 (16%) cases in the database. The handwritten medical records were manually entered into the study-specific database. Instructions for each variable guided database entry. The database fields had fixed boundaries making it impossible to enter ambiguous values, for example, numbers in text fields and numbers outside what is possible for a specific variable. The database also consisted of multiple-choice options from drop-down menus to guide database entry and ensure data quality. The variables sex, age, day of the week, time of day, ESS code, triage level colour, and destination were collected from the handwritten medical records. All database entries included personal identity numbers, which allowed correct linkage for every case. Follow-up data were both digitally and manually extracted from the hospital-specific digital medical record system (Klinisk Portal). The hospital medical record system automatically retrieves vital statistics, such as death, from the Swedish population register database. Chart review was performed by the first author.

### Outcome measures

The study focused on four outcome measures, ED visits, admissions to in-hospital care, intensive care units, and mortality, all within seven days, among patients who were not conveyed to the ED by the EMS.

### Data analysis

Before analysis, 30% of the manually entered data were randomly checked by the first author and administrators at the University Health Care Research Centre. Each handwritten record was compared with the previously entered data in the digital database. This was performed to ensure that data from the handwritten non-conveyance documents had been correctly transferred to the database. Database entry errors were < 0.25%. The variable ‘sex’ had 30 missing values, and the variable ‘time of day’ had 60 missing values. Categorical variables were described as numbers and percentages and were analysed with the chi-square test and proportion test. Continuous variables were described as the medians and interquartile ranges and were assessed with Mann–Whitney U tests and two-sample t tests. Univariable logistic regression was used to analyse the relationships between the included variables and the outcome measures. All variables except destination and ESS triage level colour were included in the regression models. Destination and ESS triage level colour were not included due to the amount of missing data. All other variables were used individually to test for linear relationships. The outcome intensive care is not included in the tables since no one received intensive care during follow up.

The data were tested by both graphical and numerical methods to assess the normality of the distributions. A p-value threshold of < 0.05 was used to test for statistical significance. Bonferroni corrections for > 5 groups were used to adjust for statistical significance. All patients who visited the ED within seven days are included in the tables and analysis regardless of the non-conveyance destination. Hospitalised patients are also included in the “ED visit” column. In total, 32 different ESS codes were used. The data were analysed using STATA 15.1 software (College Station, Texas, USA: Stata Corporation).

## Results

### Patient, assignment characteristics and outcomes

**Fig. 1:**
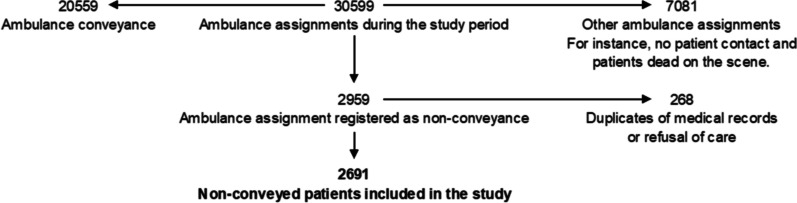
Flowchart of patients who were not conveyed during the study period

In total, 2691 non-conveyance assignments were recorded in the studied region during the study period (Fig. 1). In 49% of these non-conveyance assignments, the patient was a female. These female patients had a median age of 53 years, while the male patients had a median age of 50 years.

The age group (65–80 year) had the highest number of non-conveyed patients. Most patients were non-conveyed on Saturdays and between 18:00–23:59. In this setting, neither the time of day nor the day of the week affected the outcomes, namely, ED visit (p = 0.67, p = 0.24), hospitalisation (p = 0.44, p = 0.52) and mortality (p = 0.58, p = 0.07) (Table 1).

**Table 1:** Patient and assignment characteristics stratified by outcome, n (%) unless otherwise stated

All patients (0–99 years)	Non-conveyed	ED visit	Hospitalised	Died
Total	2691 (100)	451 (100)	137 (100)	18 (100)
*Sex*				
Male	1344 (51)	226 (50)	63 (46)	10 (56)
Female	1317 (49)	223 (50)	74 (54)	8 (44)
*Age*				
Median (Q1–Q3)	51 (25–73)	59 (26–78)	77 (58–86)	87 (80–91)
Male	50 (25–72)	57 (25–78)	71 (49–82)	84 (71–90)
Female	53 (26–77)	66 (32–81)	80 (63–87)	89 (85–92)
*Age group*				
0–10 years	271 (10)	63 (14)	13 (9)	0 (0)
11–17 years	115 (4)	18 (4)	2 (1)	0 (0)
18–30 years	471 (18)	43 (10)	2 (1)	0 (0)
31–45 years	329 (12)	47 (10)	8 (6)	0 (0)
46–64 years	492 (18)	69 (15)	18 (13)	0 (0)
65–80 years	575 (21)	110 (24)	41 (30)	5 (28)
> 80 years	438 (16)	101 (22)	53 (39)	13 (72)
*Day of week*				
Monday	390 (15)	73 (16)	18 (13)	4 (22)
Tuesday	387 (14)	64 (14)	21 (15)	1 (6)
Wednesday	350 (13)	49 (11)	27 (20)	1 (6)
Thursday	351 (13)	54 (12)	14 (10)	3 (17)
Friday	375 (14)	64 (14)	18 (13)	1 (6)
Saturday	450 (17)	81 (18)	18 (13)	4 (22)
Sunday	388 (14)	66 (15)	21 (15)	4 (22)
*Time of day*				
00:00–05:59	504 (19)	75 (17)	21 (15)	3 (17)
06:00–11:59	530 (20)	86 (19)	23 (17)	5 (28)
12:00–17:59	732 (28)	140 (31)	49 (36)	4 (22)
18:00–23:59	869 (33)	150 (33)	44 (32)	6 (33)

In the adult population, “non-specific symptoms, malaise” was the most commonly used ESS code. Less than 2% of the adult patients who were not conveyed were assigned an orange or red ESS triage level colour. However, 29% of the adult patients were not assigned an ESS triage level colour by the nurse responsible for the non-conveyance decision (Table 2).

**Table 2:** ESS codes and triage level colours for adults and children, stratified by outcome, n (%)

	Non-conveyed	ED visit	Hospitalised	Died
*ESS codes*				
Adults (18–99 years)	2305 (100)	370 (100)	122 (100)	18 (100)
53 - Non-specific symptoms, malaise	345 (15)	66 (18)	34 (28)	5 (28)
6 - Abdomen, flank or groin pain	173 (8)	41 (11)	16 (13)	2 (11)
11 - Vertigo, balance problems	97 (4)	18 (5)	6 (5)	0 (0)
12 - Neurological problems	27 (1)	10 (3)	5 (4)	0 (0)
4 - Breathing difficulties	136 (6)	21 (6)	6 (5)	2 (11)
30 - Injury, head/neck, strangulation, teeth	52 (2)	12 (3)	4 (3)	0 (0)
14 - Back or neck pain	57 (2)	7 (2)	4 (3)	0 (0)
47 - Fever, infection	59 (3)	14 (4)	5 (4)	0 (0)
20 - Loss of consciousness	74 (3)	9 (2)	4 (3)	1 (6)
50 - Hypoglycaemia	84 (4)	2 (< 1)	0 (0)	0 (0)
15 - Extremity problems/pain	36 (2)	14 (4)	4 (3)	0 (0)
5 - Chest pain	99 (4)	11 (3)	2 (2)	0 (0)
34 - Injury, legs/lower extremities	49 (2)	10 (3)	0 (0)	0 (0)
16 - Urinary problems/pain	20 (< 1)	9 (2)	3 (2)	0 (0)
40 - Intoxication	62 (3)	6 (2)	1 (< 1)	0 (0)
9 - Seizures, epilepsy	38 (2)	5 (1)	3 (2)	0 (0)
All other ESS codes	342 (15)	49 (13)	2 (2)	0 (0)
ESS code not stated	556 (24)	66 (18)	23 (19)	8 (44)

	Non-conveyed	ED visit	Hospitalised	Died
Children (0–17 years)	386 (100)	81 (100)	15 (100)	0 (100)
153 - Non-specific symptoms, worried parents	12 (3)	4 (5)	2 (13)	0 (0)
109 - Seizures, epilepsy	20 (5)	5 (6)	2 (13)	0 (0)
154 - Fever of unclear origin	28 (7)	4 (5)	1 (7)	0 (0)
104 - Breathing difficulties	41 (11)	10 (12)	1 (7)	0 (0)
130 - Injury, head/neck, strangulation, teeth	24 (6)	4 (5)	0 (0)	0 (0)
All other ESS codes	149 (39)	27 (33)	4 (27)	0 (0)
ESS code not stated	112 (29)	27 (33)	5 (33)	0 (0)

	Non-conveyed	ED visit	Hospitalised	Died
*ESS triage level colour*				
Adults (18–99 years)				
Green	1172 (51)	193 (52)	71 (58)	9 (50)
Yellow	416 (18)	98 (26)	27 (22)	2 (11)
Orange/Red	46 (2)	3 (< 1)	1 (< 1)	0 (0)
ESS triage level colour not stated	671 (29)	76 (21)	23 (19)	7 (39)
Children (0–17 years)				
Green	194 (50)	31 (38)	3 (20)	0 (0)
Yellow	52 (13)	19 (23)	5 (33)	0 (0)
Orange/Red	0 (0)	0 (0)	0 (0)	0 (0)
ESS triage level colour not stated	140 (36)	31 (38)	7 (47)	0 (0)

For children, breathing difficulties and fever of unclear origin were the most commonly used ESS codes. The most common ESS triage level colours assigned to both children and adults were green and yellow. More children than adults did not have a documented ESS triage level colour.

The most common non-conveyance destination for both children and adults was self-care (> 50%), followed by primary health care and the ED with modes of transport other than an ambulance. For adult patients, 30% had primary health care, and 19% had the ED via another mode of transportation as their assigned non-conveyance destination. The proportions of assigned non-conveyance destinations were approximately the same for children and adults (Table 3).

**Table 3:** Non-conveyance destination and patient outcomes, stratified by outcome, n (%)

	Non-conveyed	ED visit	Hospitalised	Died
(Adults, 18–99 years)	2305 (100)	370 (100)	122 (100)	18 (100)
*Destination*				
Self-care	746 (32)	45 (12)	19 (16)	5 (28)
Primary health care	441 (19)	55 (15)	18 (15)	1 (6)
Emergency department	275 (12)	136 (37)	45 (37)	3 (17)
Not stated	843 (37)	134 (36)	40 (33)	9 (50)

	Non-conveyed	ED visit	Hospitalised	Died
(Children, 0–17 years)	386 (100)	81 (100)	15 (100)	0 (100)
*Destination*				
Self-care	128 (33)	13 (16)	2 (13)	0 (0)
Primary health care	55 (14)	7 (9)	3 (20)	0 (0)
Emergency department	54 (14)	33 (41)	6 (40)	0 (0)
Not stated	149 (39)	28 (35)	4 (27)	0 (0)

### Patients who visited the emergency department

When all non-conveyed patients were considered, there was no difference with respect to the number or distribution of male and female patients who visited the ED within seven days (p = 0.88). Among the patients who visited the ED within seven days, female patients were nine years older than male patients, and the difference was significant (p = 0.02) (Table 1).

For both children and adults, patients given a yellow ESS triage level colour were more likely to have a registered ED visit within seven days than all other ESS triage level colours (p < 0.01).

Among adult patients who visited the ED after a non-conveyance decision was made, non-specific symptoms with malaise; abdominal, flank or groin pain; and breathing difficulties were the three most commonly used ESS codes. Approximately 19% of the patients who were categorised as having non-specific symptoms and malaise (ESS code 53) subsequently visited the ED (Table 2). The majority (78%) of those patients were assigned the destination of primary health care or the ED via another mode of transportation (Table 3).

Breathing difficulties were the most commonly used ESS code for patients under the age of 18 who visited the ED after the non-conveyance decision was made (Table 2). In total, 17% (children, 21%; adults, 16%) of patients visited the ED within seven days. 8% of the adult patients who were advised to continue self-care at home or to make an appointment with primary health care services visited the ED within seven days. Among children, 11% visited the ED after being advised to continue self-care or seek primary health care (Table 3). For all patients who were advised to go to the ED via another mode of transportation, nearly half (49%) did not have a registered ED visit within seven days.

### Patients who were hospitalised

Among the patients who were hospitalised within seven days of the non-conveyance decision, there was a statistically significant age difference of nine years between the male and female patients; the female patients were older (p < 0.03). Older age was associated with the outcome of hospitalisation within seven days after the non-conveyance decision for both male and female patients (p < 0.01) (Table 1). Older age was the strongest predictor of hospitalisation.

The adjusted odds ratios were 1.03 [95% confidence interval (CI) 1.02 to 1.05] among females and 1.02 [95% confidence interval (CI) 1.01 to 1.03] among males. For children, the yellow ESS triage level colour was the only triage level associated with a higher degree of hospital admission within seven days (p < 0.01). For adult patients, there was no association between ESS triage level colour and the proportion of patients hospitalised within seven days (p = 0.71). Among the adult patients who were hospitalised within seven days after the non-conveyance decision, non-specific symptoms with malaise and abdominal, flank or groin pain were the two most commonly used ESS codes (Table 2). Among the adult patients who were assigned the destination of the ED via another mode of transport, 15% were hospitalised. Among children, the proportion was 6%. Among all adult and paediatric patients who were advised to continue self-care or seek primary health care, approximately 3% were hospitalised within seven days after the non-conveyance decision. Less than 4% of all non-conveyed children were hospitalised. When the children who were advised to go to the ED via another mode of transport were excluded, approximately 2% of the children were hospitalised. Of the non-conveyed adult patients, 5% were hospitalised (Table 3).

### Patients that received intensive care

No patients received intensive care within seven days after the non-conveyance decision.

### Patients who died

In total, 18 patients (0.7%) died within one week after the non-conveyance decision. The deceased patients were between the ages of 65–96 years, with a median age of 87 years. There was no statistically significant age difference between male and female patients who died within seven days after the non-conveyance decision (p < 0.54). There was a positive linear relationship between age and mortality among both female and male patients within seven days after the non-conveyance decision (p < 0.01) (Table 1).

Among the patients who died within seven days, non-specific symptoms with malaise; abdominal, flank or groin pain; and breathing difficulties were the most common ESS codes. Eight (44%) of the patients who died were not assigned an ESS code, and seven (39%) were not assigned an ESS triage level colour (Table 2). Of the patients who died, 14 (78%) had a nurse or physician who was present when the non-conveyance decision was made or who was responsible for continuing care. Six of those 14 patients had a documented palliative or end-of-life decision and care plan. One of the deceased patients received another ambulance assessment the same day, which led to ambulance transport to the ED, admittance to an in-hospital ward, and death within seven days. For the remaining three patients who died within seven days after the non-conveyance decision, the documentation was inconclusive or missing. Four of the patients visited the ED and were admitted to an in-hospital ward before their death.

**Fig. 2:**
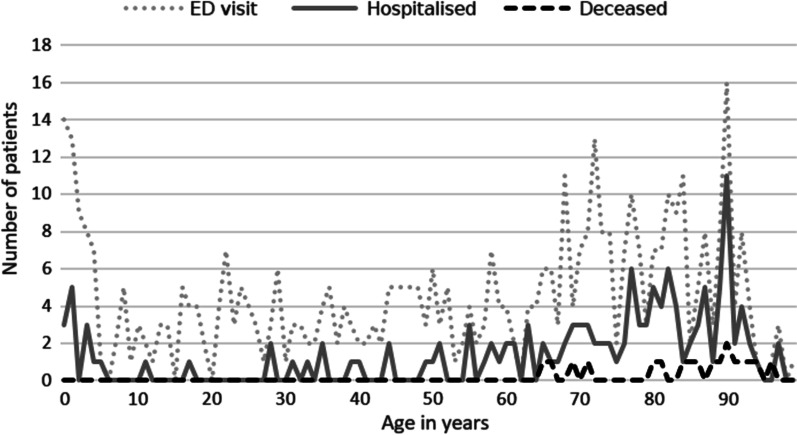
Patients visiting the ED, being hospitalised, or dying within one week after the non-conveyance decision

All outcome measures are graphically shown in Fig. 2.

## Discussion

This study focused on the four outcomes, namely, ED visit, hospitalisation, intensive care, and death within seven days after the non-conveyance decision.

Older age was the only variable associated with a higher risk for all outcomes. This result is similar to that of previous studies showing that age is a predictor of health-related outcomes [23, 24]. Age should therefore be used as a predictor for adverse outcomes and incorporated into the regional non-conveyance guidelines to minimise risks.

Previous studies have also shown that patients with vague or unspecific complaints have a higher risk of adverse outcomes [2, 11]. This finding needs to be considered since non-specific symptoms and malaise (ESS code 53) was the most commonly used triage code assigned to the patients in the studied region.

### Patients who visited the emergency department

Approximately one in five patients categorised as having a non-specific complaint visited the ED within seven days. It was also more likely that a patient assigned a yellow ESS triage level colour had a registered ED than a patient assigned a different triage level colour. The association between triage levels and the studied outcomes was inconclusive regarding the predictive value for patients triaged with non-acute complaints. This was also shown by Ivic et al., who found that more than one-third of the patients presenting with unspecific complaints had a serious underlying condition. These patients with unspecific complaints also received a low triage status [25]. Therefore, further research is needed that focuses on the association between triage levels and adverse outcomes.

### Patients who were hospitalised

5% of all non-conveyed patients were hospitalised within seven days. If patients who were advised to visit the ED via another means of transportation were excluded, 3% of the patients who were not conveyed in the studied region were hospitalised. This is at the low end of the national and international rates, which range from 5 to 12% [2, 4, 13, 26].

Older age was shown to be associated with a higher risk of hospitalisation for non-conveyed patients in the present study. There was also a nine-year age difference between hospitalised male and female patients. Previous studies have demonstrated that there is no age difference between non-conveyed male and female patients. However, as patients become older, there seems to be an increasing number of female patients who are non-conveyed [11, 27]. Whether these differences between male and female patients have any clinical relevance needs to be further investigated.

### Patients that received intensive care

No patients received intensive care within seven days after being non-conveyed in the studied region. Paulin et al. described that 0.3% of the patients in the Finnish non-conveyance system studied received intensive care within 24 h [28]. Few patients received intensive care, and several factors contributed to the decision to admit a patient to an intensive care unit [29]. Nevertheless, a significant need for intensive care following non-conveyance in the studied region was not found.

### Patients who died

Less than 1% (0.7%) of patients that were non-conveyed in the studied region died within seven days. This is at the low end of previously published mortality rates for non-conveyed patients (0.3–6.1% died within 72 h) [15]. Mortality was also shown to increase with age for patients not conveyed in the studied region. The finding that age can be a predictor of adverse outcomes has been described previously [11, 23, 30]. However, it is important to describe why patients are dead at follow-up since it may be appropriate and ethical for some patients to not be conveyed to the ED but rather to receive continued care at their own home or a care facility. In the present study, 14 (78%) of the patients who died within seven days had a documented palliative or end-of-life decision and care plan. Expected and unexpected deaths following non-conveyance need to be taken into account so that guidelines and decision support systems are based on true emergencies.

It is important to be aware of the risks when comparing different non-conveyance systems and their outcome measures due to differences in health care structures, competencies, and guidelines. Existing outcome measures, such as the rate of non-conveyance, ED visits, hospitalisation, intensive care, and death, also need to be reported in a more uniform manner to make it more feasible to compare different non-conveyance systems.

As a first step in the continuing efforts to create more precise and robust non-conveyance guidelines, we propose using a differentiated algorithm depending on the patient’s age. However, other variables have also been shown to be predictive. Some ESS codes may also be associated with greater risks [23]. A more liberal approach to continued or follow-up health care contact could potentially reduce the risk of unnecessary harm to an acceptable minimum [31].

## Conclusions

Today, there is no uniform definition of what a patient-safe system is. Few patients compared to previous studies were admitted to the hospital, intensive care or died within seven days. This study contributes new insights that can be used to improve the non-conveyance guidelines and minimise the risk of patient harm. Of all the studied variables, age was the only variable associated with the studied outcome measures (emergency department visits, hospital admission, intensive care and mortality, all within seven days).

## Limitations

This study’s main weakness was the amount of missing data, especially for the destination and ESS triage level colour. Missing data have been shown to be a problem in other studies [2, 32, 33]. It is unclear why there were so much missing data for both the ESS triage level colour and destination. It could be due to a lack of interest or because the ambulance personnel did not understand why this information was important to document. The amount of missing data for the ESS triage level colour and destination made it inappropriate to perform additional in-depth analyses for those variables. Missing data may lead to misinterpretations. Missing data could also be related to the non-electronic medical record system used by the EMS in the studied region. Incomplete triage of non-conveyed patients according to the guidelines was also reported by Magnusson et al. [2]. An effective way of improving adherence, complete documentation and potential patient outcomes is to incorporate the guidelines and documentation into digital solutions [32, 33]. The reasons for and effect of missing data in this context require further investigation. Since protocols and practices differ between contexts and change over time, the results are time- and context dependent. Both planned and unplanned ED visits were recorded. According to the non-conveyance definition, both planned and unplanned ED visits can be a consequence of non-conveyance. However, for clarity, it would have been better if patients who were told to go to the ED by themselves had been reported separately as well.

## Supplementary Information

**Additional file 1:** **The regional non-conveyance checklist**. Algorithm for referral to appropriate level of care for non-conveyed patients in the County Council of Örebro.

**Additional file 2:** **Exclusion criteria for non-conveyance**. Exclusion criteria include mental status, decision-making capability, indication of acute serious illness or acute serious deterioration of chronic illness, and monitoring or treatment needs during transport.

## Data Availability

The datasets generated and analysed during the current study are not publicly available due to patient privacy concerns. The datasets used and analysed during the present study are available from the corresponding author (EH) on reasonable request.

## Abbreviations

EMS: Emergency medical services
ESS: Emergency signs and symptoms
RETTS: Rapid Emergency Triage and Treatment System
