# Supplementary figures and images for: Outcomes in patients not conveyed by emergency medical services (EMS): a one-year prospective study
Source: Scand J Trauma Resusc Emerg Med. 2022 Jun 13;30:40. doi: 10.1186/s13049-022-01023-3 (PMC9195370; doi:10.1186/s13049-022-01023-3)

**Additional file 1.** The regional non-conveyance checklist.


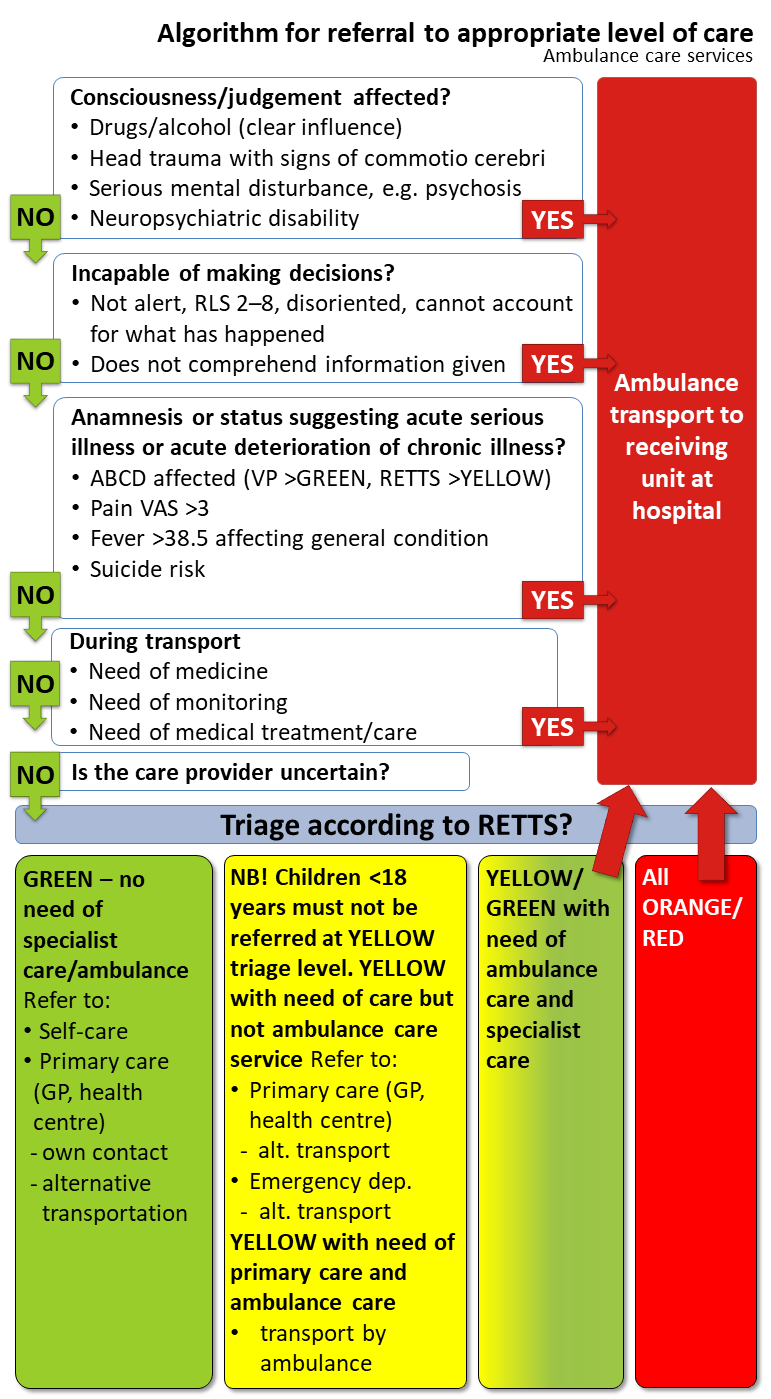

Supplement: Supplementary file 1 — Additional file 1: The regional non-conveyance checklist. Algorithm for referral to appropriate level of care for non-conveyed patients in the County Council of Örebro. [file 13049_2022_1023_MOESM1_ESM.docx]
