# Supplementary material for: Outcomes in patients not conveyed by emergency medical services (EMS): a one-year prospective study
Source: Scand J Trauma Resusc Emerg Med. 2022 Jun 13;30:40. doi: 10.1186/s13049-022-01023-3 (PMC9195370; doi:10.1186/s13049-022-01023-3)
Supplement: Supplementary file 2 — Additional file 2: Exclusion criteria for non-conveyance. Exclusion criteria include mental status, decision-making capability, indication of acute serious illness or acute serious deterioration of chronic illness, and monitoring or treatment needs during transport. [file 13049_2022_1023_MOESM2_ESM.docx]

**Additional file 2.** Exclusion criteria for non-conveyance

**For non-conveyance, all of the criteria below must be fulfilled.**

**1. Is the patient mentally affected? – If so, non-conveyance is not an option.**

Examples of causes for being mentally affected:

**a.** Influence of drugs/alcohol (= clearly under the influence)

**b.** Head trauma with signs of concussion (suspected commotio cerebri)

**c.** Serious mental disturbance (e.g. psychosis)

**d.** Neuropsychiatric disability.

**2. Is the patient capable of making decisions? – If not, non-conveyance is not an option.**

**a.** Children <18 years are only considered capable of making decisions in the presence of their parent or guardian.

**b.** To be considered capable of making decisions, the subject must be alert, RLS 1, not somnolent, not disoriented, and be able to account for what has happened.

**c.** To be considered capable of making decisions, the subject has to be able to understand the information given – there must not be any language problems concerning either language comprehension as such or other reasons for difficulties in communicating, such as e.g. dementia or psychiatric influence.

**3. Anamnesis and status according to the treatment handbook.**

**a.** All anamnesis data indicating acute serious illness or acute serious deterioration of chronic illness are counter-indicative of non-conveyance.

**b.** Serious impact on vital functions/parameters in ABCDE is counter-indicative of non-conveyance (the patient must be triaged green according to vital parameters).

**c.** Serious pain VAS >3 counter-indicates non-conveyance.

**d.** Fever >38.5°C with impact on general condition or shivering fits counter-indicate non-conveyance.

**4. Need of administration of medicine, monitoring or medical treatment/care**

**during transport? – If so, non-conveyance is not an option.**

**a.** Medicine: e.g. oxygen, pain killers, infusion.

**b.** Monitoring during transport: e.g. EKG, respiratory rate, pulse oximetry, pulse and blood pressure, level of consciousness.

**c.** Medical treatment/care: e.g. management of airway, CPR, immobilisation
